# Models of intestinal infection by *Salmonella enterica*: introduction of a new neonate mouse model

**DOI:** 10.12688/f1000research.8468.1

**Published:** 2016-06-24

**Authors:** Marc Schulte, Michael Hensel

**Affiliations:** 1 Department of Microbiology, University of Osnabrück, Osnabrück, Germany

**Keywords:** Salmonella, intestinal inflammation, neonate mouse model, infection

## Abstract

*Salmonella enterica* serovar Typhimurium is a foodborne pathogen causing inflammatory disease in the intestine following diarrhea and is responsible for thousands of deaths worldwide. Many *in vitro* investigations using cell culture models are available, but these do not represent the real natural environment present in the intestine of infected hosts. Several *in vivo* animal models have been used to study the host-pathogen interaction and to unravel the immune responses and cellular processes occurring during infection. An animal model for *Salmonella*-induced intestinal inflammation relies on the pretreatment of mice with streptomycin. This model is of great importance but still shows limitations to investigate the host-pathogen interaction in the small intestine *in vivo*. Here, we review the use of mouse models for *Salmonella* infections and focus on a new small animal model using 1-day-old neonate mice. The neonate model enables researchers to observe infection of both the small and large intestine, thereby offering perspectives for new experimental approaches, as well as to analyze the *Salmonella-*enterocyte interaction in the small intestine *in vivo*.

## Introduction

The Gram-negative Enterobacteriaceae *Salmonella enterica* are among the main causes of bacterial gastrointestinal infections of millions of humans and animals around the world every year. The highest infection risk is oral ingestion of contaminated food or water often associated with insufficient hygiene conditions, but even industrial countries are not safe from infections ^1, 2^. About 2600 serovars of *S. enterica* are known, and serovars pathogenic to humans cause typhoidal and non-typhoidal forms of disease. The systemic disease enteric typhoid fever is caused by the human-restricted typhoidal serovars Typhi and Paratyphi A and is associated with high mortality if not treated by antibiotics. Non-typhoidal serovars, predominantly Enteritidis and Typhimurium, can infect a broad range of animals and humans, causing an acute self-limiting gastroenteritis associated with intestinal inflammation and diarrhea. This gastroenteritis is usually self-limiting without serious complications. However, systemic spread of non-typhoidal *S. enterica* may occur in infants, in the elderly, or in people with underlying infections or immunodeficiency (for example, due to HIV infection) and result in a more severe disease outcome. Persistent infections caused by non-typhoidal *Salmonella* are poorly investigated, and the prevalence of long-term non-typhoidal *Salmonella* carriers is not known ^3– 6^.

*Salmonella* pathogenicity is mediated mainly by horizontally transferred chromosomal regions, encoding sets of virulence factors enabling the pathogen to successfully infect and colonize its host. The role in pathogenesis and the molecular functions of these so-called *Salmonella* pathogenicity islands (SPIs) are partly understood, but many functions remain to be resolved ^7^. The SPI1 encodes a type III secretion system (T3SS) and the associated effector proteins, which can be injected into the host target cell (for example, epithelial cells) and thereby promote pathogen-induced internalization by non-phagocytic cells ^8– 11^.

The invasion of non-phagocytic cells by *Salmonella* and intracellular proliferation has been studied in much detail by using *in vitro* models ^12, 13^. Also, enterocyte invasion in different *ex vivo* tissue explants has been observed ^14– 16^. Nevertheless, these models bear limitations in studying the host-pathogen interaction in the natural anatomical environment, especially with respect to hallmarks of *Salmonella* pathogenesis – the invasion of polarized epithelial cells, intracellular survival, and formation of microcolonies.

Murine infection models are attractive, since mice can be genetically manipulated. A previously developed mouse model for intestinal inflammation by *S. enterica* was based on antibiotic pretreatment of adult mice to reduce intestinal microbiota ^17^. Here, we discuss a new infection model deploying 1-day-old neonate mice that allows the investigation of *Salmonella* enterocyte invasion, intracellular proliferation, and microcolony formation *in vivo* without pretreatment by antibiotics ^18^.

## Animal models of non-typhoidal *Salmonella* infections

The disease outcome of *S. enterica* infection, that is intestinal inflammation and diarrhea, or systemic infection with colonization of other organs is dependent on the host susceptibility and the serotype of the pathogen ^19^. In humans or cattle, *S. enterica* serovar (sv.) Typhimurium induces enterocolitis, resulting in intestinal inflammation and diarrhea, whereas infected mice present no intestinal inflammation because of intrinsic resistance ^20^. Nevertheless, certain mouse strains with defects in genes encoding SLC11A1 (previously named NRAMP1) develop a typhoid-like disease, similar to human infection with typhoidal serovars ^21^. In infected susceptible mice, *S. enterica* sv. Typhimurium penetrates the epithelial barrier by invasion of microfold cells (M cells) or transport via dendritic cells ^22– 24^. M cells are specialized epithelial cells located in Peyer’s patches, which are organized lymphoid regions of the intestine. M cells phagocytose and transport antigens and bacteria to immune cells present in Peyer’s patches ^25^. *Salmonella*-susceptible mice develop systemic infection after colonization of Peyer’s patches and mesenteric lymph nodes following spread to liver and spleen ^26, 27^. Owing to this pathogenesis and the absence of an appropriate small animal model, detailed analyses of *Salmonella* gastroenteritis were not possible. Earlier work investigated *Salmonella*-induced diarrhea in Rhesus monkeys ^28^. As an alternative, infection of ligated murine and rabbit ileal loops was used as a model ^22, 29^. Furthermore, a bovine infection model was established that allowed the identification of certain *Salmonella* associated factors, such as the SPI1-T3SS, needed to induce enterocolitis ^20^. In addition, bovine ligated ileal loops infected with *Salmonella* were investigated ^16, 30, 31^. However, the use of large animals for infection causes technical limitations, and only restricted investigation of the role of the host in the host-pathogen interaction is possible. Hence, many features of *Salmonella* intestinal pathogenesis were analyzed by using tissue culture or intestinal organ culture ^32^.

Oral application of the antibiotic streptomycin makes mice more susceptible to infection with *Salmonella* ^33– 35^. This effect was ascribed to removal of commensal intestinal microbes by streptomycin ^36, 37^. Based on these observations, a mouse model of oral infection of 6- to 8-week-old mice after pretreatment with streptomycin was established ^17^, and this enabled the investigation of *Salmonella*-induced colitis in small animals. The pathogenesis of colitis caused by *S. enterica* sv *.* Typhimurium in streptomycin-pretreated mice showed many similarities to the human infection and its pathology is highly dependent on function of the SPI1-T3SS. With a knockout mouse strain that lacks all lymph nodes and organized gut-associated lymphatic tissues, it was shown that Peyer’s patches and mesenteric lymph nodes are not necessary for the induction of colitis ^17^.

However, there are some differences between the bovine and human infections and the infection in streptomycin-pretreated mice with regard to the intestinal location of the symptoms. Streptomycin-pretreated mice show inflammation of the cecum and the colon, whereas both the small and large intestine are affected in infections of cattle or humans ^20, 38^. Furthermore, the infection of rabbits, calves, and primates is often accompanied by massive luminal fluid secretion; however, streptomycin-pretreated mice do not show this phenomenon ^21^. Translocation of *Salmonella* over the colonic epithelium in the absence of intracellular proliferation ^39^ as well as enterocyte invasion and presence of *Salmonella* in the *lamina propria* in the mouse large intestine was demonstrated by using streptomycin-pretreated mice ^40– 42^. However, streptomycin-pretreated adult animals did not allow investigation of the infection process of the small intestinal epithelium, including invasion into polarized epithelial cells and intracellular survival.

To understand the role of bacterial as well as host factors for pathogenesis, it is of great importance to analyze the interaction of *Salmonella* with host cells within their natural environment. These factors, facilitating enterocyte invasion but also intraepithelial proliferation resulting in formation of intraepithelial bacterial colonies, remained undefined.

## A new neonate mouse model for *Salmonella*

A small animal model using 1-day-old C57BL/6 mice was recently reported ^18^ that may serve as an attractive alternative to the use of streptomycin-pretreated mice. An investigation of oral *Salmonella* infection of neonate and adult mice was accomplished, revealing age-dependent differences in intestinal colonization, mucosal translocation, and systemic spread. The work demonstrated rapid colonization of the small intestine and the colon of neonate mice, as opposed to adult animals as well as efficient entry of *Salmonella* into intestinal epithelial cells, followed by bacterial proliferation and formation of intraepithelial microcolonies.

**Figure 1.**
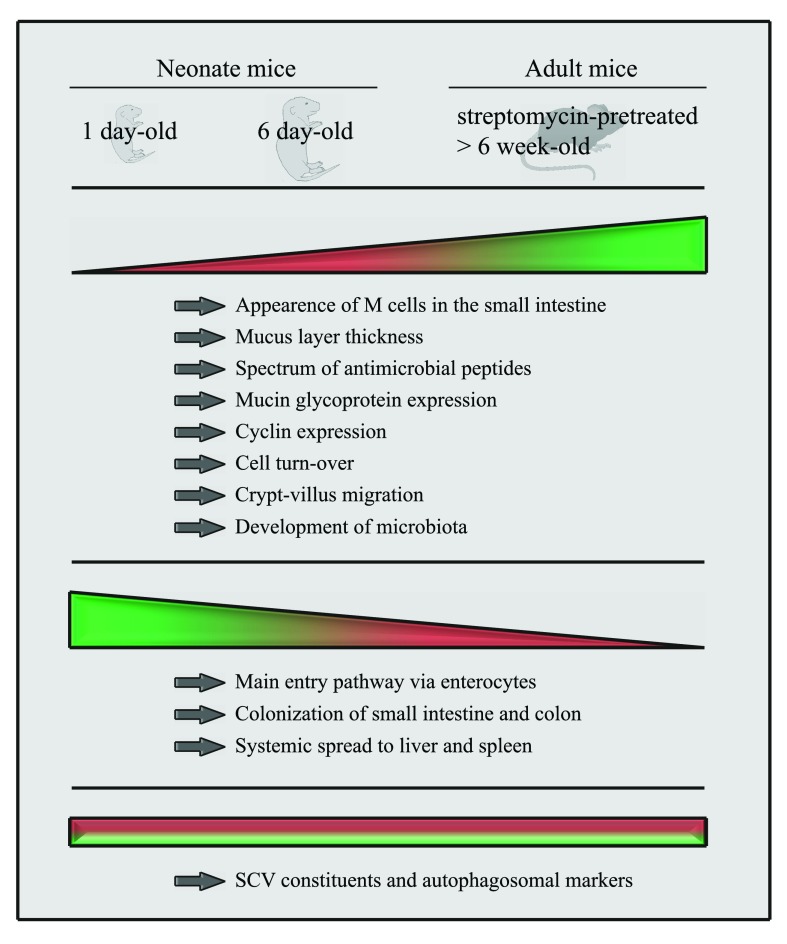
Age-dependent differences in intestinal colonization, mucosal translocation, and systemic spread. A comparison of 1-day-old, 6-day-old, and streptomycin-pretreated 6-week-old C57BL/6 mice shows many differences in intestinal colonization, mucosal translocation, and systemic dissemination in comparison with other organs after oral infection with *Salmonella enterica* serovar Typhimurium. Shading indicates no or low (red) and fully developed (green) features. M cell, microfold cell; SCV, *Salmonella*-containing vacuole.

The penetration of the mucosal barrier was dependent on enterocyte invasion and led to systemic spread to liver, spleen, and mesenteric lymph nodes. Without a functional SPI1-T3SS, systemic spread of *Salmonella* was largely abolished. Enterocytes were infected by wild-type, but not SPI1-T3SS-deficient, *Salmonella*. The major entry pathway for bacterial translocation is dependent on M cells ^23^, but this cell population appears only after the neonatal period. It was shown that the expression of genes of differentiated M cells (for example, Spi-B and Ccl9) was very low in epithelial cells of neonate mice. Furthermore, no M cell markers like glycoprotein 2, Ulex europaeus agglutinin, or Ccl9 were found by immunostaining intestinal tissue. That shifts the major port of entry to enterocyte invasion and therefore is SPI1-T3SS dependent and M cell independent, whereas intestinal colonization and systemic spread in adult streptomycin-pretreated mice is SPI1 T3SS independent because of the presence of M cells. Moreover, the invasion of epithelial cells by *Salmonella* leads to intraepithelial proliferation and formation of intraepithelial microcolonies that arise from a single event of bacterial invasion. In addition, cells infected with *Salmonella* appear morphologically intact despite invasion and proliferation ^18^. Differences and similarities of *Salmonella* infection models of neonate and adult mice are summarized in Figure 1.

Low expression of mucin glycoproteins and lower thickness of the mucus layer were measured in neonate mice. Neonates showed a lower antimicrobial peptide repertoire in accordance with previous reports ^43^. In contrast, adult animals exhibit the synthesis of various mucin glycoproteins, an enhanced thickness of the mucus layer, and antimicrobial peptides to create a strong barrier against pathogens ^44, 45^. A further difference between the epithelium of neonate and adult mice is an altered cyclin expression, resulting in a lower crypt to villus migration and minimal turnover of enterocytes, whereas enterocytes of adult animals show a constant renewal ^46^. Therefore, the intestinal epithelial cells of neonate mice stay longer at the same position. It is speculated that the extended lifetime of epithelial cells and minimal expression of mucin glycoproteins and antimicrobial peptides as well as a reduced thickness of the mucus layer allow *Salmonella* to invade epithelial cells, to proliferate, and finally to form microcolonies. Another important difference is the lack of an established intestinal microbiota in neonate mice. A developed and diverse enteric microbiota is considered as a key factor of colonization resistance against *Salmonella* in adult mice. In the streptomycin pretreatment model, this colonization resistance is overcome by antibiotic elimination of a major fraction of the microbiota.

An innate immune response was further activated via Toll-like receptor (TLR) stimulation by intraepithelial, but not by non-invasive, extracellular *Salmonella*. After infection of neonate mice by wild-type *Salmonella*, a time-dependent increase in Cxcl2 and Cxcl5 mRNA expression was observed while mRNA expression was strongly reduced in enterocytes of adult mice isolated at day 4 after infection. This underlines the requirement of enterocyte invasion for the innate immune stimulation. Additionally, a large number of additional genes involved in metabolism, communication processes, and cellular responses were induced after infection. In the absence of innate immune receptors like TLR4, TLR2, TLR5, TLR9, MyD88, Unc93B1, and Nod2, the expression of Cxcl2 and Reg3γ mRNA by epithelial cells was severely reduced. Even in the absence of the most effective innate immune receptor, TLR4, an intestinal colonization of *Salmonella* as well as enterocyte invasion and spread to systemic organs were observed. Compared with infection of adult hosts, neonatal intestinal tissue remained largely intact in terms of histopathological parameters and epithelial barrier integrity.

There are some important similarities such as the constituents of the mature *Salmonella*-containing vacuole as well as autophagosomal factors that are expressed by both neonatal and adult epithelial cells. This may enable the future investigation of the intracellular lifestyle of *Salmonella* during the initial phase of infection.

## Advantages, limitations, and future perspectives

In contrast to other infection models (for instance, the bovine host), the mouse is amenable to efficient genetic manipulation and therefore offers the opportunity to analyze the host-pathogen interaction with both genetically altered pathogen and host ( Table 1). Owing to the absence of M cells in the neonate host, *Salmonella-*enterocyte interaction as well as invasion of polarized epithelial cells, intraepithelial proliferation, and the formation of microcolonies can be observed in their natural anatomical environment, which is a requirement for understanding the contribution of bacterial virulence factors. This may enable researchers to characterize the early steps in *Salmonella* pathogenesis and to discover the functional role of effector proteins injected into the host cell via secretion systems. The analysis of SPI1-T3SS and SPI2-T3SS effector translocation can perhaps be followed by live cell imaging by using effector proteins fused to self-labeling enzyme tags. Moreover, new approaches to investigate the innate immune response of the host are made possible. The morphological features of cells infected with *Salmonella* are largely unaltered and, despite an innate immune response, the severe damage of the epithelium observed in adult animals is absent in infected neonates.

**Table 1.** Advantages and limitations of the neonate mouse model for investigation of *Salmonella*-enterocyte interaction *in vivo*.

Advantages	Limitations
No antibiotic pretreatment of the host required	Small animal size, special care for handling
Invasion of small intestine enterocytes by *Salmonella*	Short time window for experiments
Intraepithelial proliferation and microcolony formation	Lack of suitable anesthetics needed for intravital microscopy
Characterization of early stages in intestinal pathogenesis	Investigation of entry via microfold cells not possible
Host cells appear morphologically intact despite invasion and intracellular proliferation	Reduced/altered microflora
Accessible to genetic manipulation of the host	Spread of the pathogen to systemic sites
Analysis of innate immune response of the host	

The neonate mouse animal model provides advantages in investigating the *Salmonella*-enterocyte interaction *in vivo*, but there are some limitations like the small animal size and a lack of suitable anesthesia needed for intravital microscopy that have to be considered. Owing to the lack of M cells, one important route of infection is not represented in the neonate model. The role of intestinal microbiota during *Salmonella* infection cannot be addressed, since the microbiome in neonates is highly reduced and distinct from the microbiota of adult individuals. Regardless, the new neonate animal model could contribute to a better understanding of the cellular processes during infection. The model allows *in vivo* analyses of both hallmarks of *Salmonella* pathogenesis: the internalization by non-phagocytic cells and the intracellular activity of *Salmonella* leading to the formation of intraepithelial microcolonies.

## Abbreviations

M cell, microfold cell; SPI, *Salmonella* pathogenicity island; T3SS, type 3 secretion system; TLR, Toll-like receptor.
